# Bayesian Revision vs. Information Distortion

**DOI:** 10.3389/fpsyg.2018.01550

**Published:** 2018-08-28

**Authors:** J. Edward Russo

**Affiliations:** ^1^SC Johnson College of Business, Cornell University, Ithaca, NY, United States; ^2^Samuel Curtis Johnson Graduate School of Management, Cornell University, Ithaca, NY, United States

**Keywords:** Bayesian calculus, connectionism, desirability bias, forecasting, information distortion, likelihood updating, rationality, statistical inference

## Abstract

The rational status of the Bayesian calculus for revising likelihoods is compromised by the common but still unfamiliar phenomenon of information distortion. This bias is the distortion in the evaluation of a new datum toward favoring the currently preferred option in a decision or judgment. While the Bayesian calculus requires the independent combination of the prior probability and a new datum, information distortion invalidates such independence (because the prior influences the datum). Although widespread, information distortion has not generally been recognized. First, individuals are not aware when they themselves commit this bias. In addition, it is often hidden in more obvious suboptimal phenomena. Finally, the Bayesian calculus is usually explained only with undistortable data like colored balls drawn randomly. Partly because information distortion is unrecognized by the individuals exhibiting it, no way has been devised for eliminating it. Partial reduction is possible in some situations such as presenting all data simultaneously rather than sequentially with revision after each datum. The potential dangers of information distortion are illustrated for three professional revision tasks: forecasting, predicting consumer choices from internet data, and statistical inference from experimental results. The optimality of the Bayesian calculus competes with people's natural desire that their belief systems remain coherent in the face of new data. Information distortion provides this coherence by biasing those data toward greater agreement with the currently preferred position—but at the cost of Bayesian optimality.

The information needed for nearly all important decisions falls into two categories, values and likelihoods. These decisions typically share two characteristics. First, both values and likelihoods are mainly subjective. Even objective information often requires a subjective evaluation of its decision impact. Second, important decisions usually involve the search for additional information that then drives the revision of the values and likelihoods. While there is no optimizing guidance for revising values, there is for likelihoods. That guidance is the Bayesian calculus^1^.

Bayesian revision combines a prior probability and the diagnostic value of a new datum (i.e., a unit of new information). The calculus of that combination requires that the prior probability and the datum contribute independently to the revised posterior probability. Unfortunately, a common phenomenon of likelihood revision can lead to the violation of that independence assumption.

This phenomenon is the predecisional distortion of information (Russo et al., 1996). It is illustrated by a study of whether to invest in a resort hotel (Russo and Yong, 2011). The sole investment criterion was risk as indexed by the probability of financial failure. Thus, the investment decision depended solely on the likelihood of failure. As information about the hotel was presented, the experimental participants revised the probability of the hotel's financial failure. That is, they repeatedly calculated a revised posterior probability after each new unit of information/datum.

The decision process, or equivalently the revision of posterior probabilities, was tracked by requiring two responses for each datum. The first was the judged diagnosticity of that datum. The second was the posterior probability, that is, the datum-driven revision of the likelihood of investing. The predecisional distortion of information is a bias in the evaluation of the new datum/information toward supporting whichever of the decision options is currently “in the lead.” Consider potential investors who are leaning toward investing based on all the data seen so far (and captured by the prior probability). Then information distortion occurs when these investors bias their evaluation of the next datum toward investing. Conversely, if the same investors had been leaning toward not investing, information distortion (ID) is manifest as a biased interpretation of the next datum toward not investing. ID is a specific, process-explicated example of the many phenomena exhibiting a confirmation bias.

The impact of the predecisional distortion of information on the Bayesian calculus is depicted in Figure 1. The left panel illustrates the independent contributions of the prior and the datum to the posterior probability. The right panel adds the biasing influence of the prior on the datum. This is the influence of the current/prior leaning toward one option on the evaluation of the next datum/information. In their study of the resort investment decision, Russo and Yong (2011) reported significant information distortion (ID). This finding accords with similar risky decisions studied by DeKay et al. (DeKay et al., 2009, 2011; Glöckner and Herbold, 2011; Miller et al., 2013).

**Figure 1:**
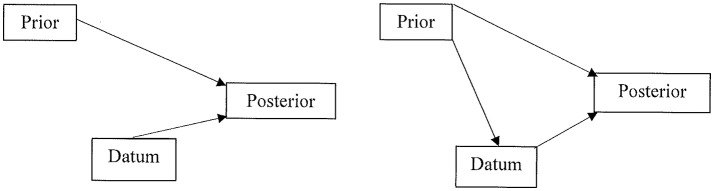
Left panel: valid Bayesian calculus with independent inputs from both the prior probability and the datum to the posterior probability. Right panel: invalid Bayesian calculus because the prior influences the perception and evaluation of the datum, thereby violating their independent contribution to the posterior probability.

The phenomenon of information distortion (ID) during the revision of probabilities raises several questions. First, is ID widespread enough to affect a substantial number of revision tasks? Second, why has this bias not been recognized (and its consequences for the validity of the Bayesian calculus not been appreciated)? Third, what can be done to eliminate ID? The remainder of this article addresses these three questions. It concludes, first, with a consideration of where ID might undermine applications of the Bayesian calculus and, second, with a comment on the frequent clash between the ideal of normative criteria and the reality of human cognition (e.g., Thaler, 1992) of which the Bayesian calculus vs. ID is only one example.

## How widespread is information distortion?

Reviews by DeKay (2015) and by Russo (2015) report the near universal presence of ID in decisions where the relevant information is acquired over time. Besides studies with college students and MTurk workers, ID has been found in decisions made by auditors, entrepreneurs, physicians, prospective jurors, and sales representatives.

In addition, ID is a systematic function of the prior commitment to the tentatively preferred course of action, as indexed by the prior probability. If that commitment is increased, ID rises in parallel (Polman and Russo, 2012).

ID is also persistent. Occasionally the new information/datum is so anti-leader that the posterior probability reflects a reversal of the leading option. In the above example, this might mean switching the tentative preference from investing to not investing. When such a preference reversal occurs, ID biases the evaluation of new information toward the new leading option, such as toward not investing. (However, see, Carlson et al., 2013, for residual traces of an initial preference).

## Why has information distortion not been recognized?

The presence of ID has gone unrecognized for multiple reasons. First, decision makers themselves are unaware that they distort new information. When ID is described to experimental subjects, their estimates of it in their own just-completed decision correlates essentially zero with their actual level of ID (Russo, 2015).

Second, sometimes ID is hidden among other biases. For example, ID is one among many possible causes of the desirability bias in which people overestimate the likelihood of a desired event (Russo and Corbin, 2016).

A third reason for the failure to detect the presence of ID may be peculiar to the canonical Bayesian setting. The familiar demonstrations of the Bayesian calculus have tended to use undistortable data, like contrastingly colored balls drawn randomly from an urn. It is impossible to distort the draw of 3 blue and 7 green balls, however strong may be the prior for one color.

In summary, the difficulty of recognizing ID in likelihood revision seems to have multiple causes. Some causes of ID seem omnipresent, such as the absence of self-awareness. Others operate only in certain situations, such as when conflated with other biases like desirability.

## Is there remediation?

Remediation can be achieved, but only partially and in select circumstances. Consider first what may be the most obvious tactic for complete elimination, paying people to be accurate and, therefore, unbiased. (Meloy et al., 2006 see also Engel and Glöckner, 2013) found that incentives increased rather than decreased ID. Further, the negative impact of money held when people were paid both for the accuracy of the decision and, more importantly, for the accuracy of the evaluation of each datum. (This anomalous result was caused by the positive mood induced by the incentive, Meloy, 2000).

A second potential path to remediation is to identify the cause of ID, which might suggest a method for its elimination. Russo et al. (2008) showed that ID is caused by the general desire for cognitive consistency (Gawronski and Strack, 2012). Specifically, decision makers want the datum (new information) to be consistent with the prior (which reflects all past information). In order to achieve greater consistency they distort the datum in the direction favored by the prior. Unfortunately, knowing that the goal of cognitive consistency drives ID does not reveal a method for ameliorating this bias besides the difficult task of increasing the tolerance for inconsistency. While methods for activating the consistency goal exist (Chaxel and Russo, 2015), those for deactivating it have proved elusive.

In spite of the failure of the above two common paths to amelioration, there have been some partial successes in reducing ID. The first relies on the simultaneous rather than sequential presentation of the data (Carlson et al., 2006). Simultaneous presentation reduces to near zero the delays between data that enable, even encourage, likelihood revision. Of course, such massed presentation does not prohibit spaced revision of the prior. Decision makers can still pause to consider the impact of each new datum before moving to the next information. Nonetheless, the simultaneous presentation of all data seems to inhibit such revision. One reason may be that the time and effort to process all the data at once are likely less than the cumulative total time and effort of several individual revisions.

A second tactic is creating precommitment to the diagnostic value of a datum. That is, each datum is evaluated prior to and, importantly, independent of a particular decision. Carlson and Pearo (2004) showed that if decision makers have knowledge of a datum outside the context of a decision, then ID is reduced almost to zero when that same datum appears during a decision.

Third, decision-making groups exhibit no ID so long as different members maintain opposing positions (Boyle et al., 2012). As long as some members favor one option while other members lean toward another option, there is sufficient debate on the pros and cons of each to suppress ID. This said, most decisions are not made in groups. Further and more worrying, once all members begins to lean toward the same option, ID grows to a level substantially above that of individuals.

In summary, ameliorative tactics are at least partially successful under some circumstances. However, no general strategy for eliminating ID has yet been devised.

## The risk of information distortion in applications of Bayesian inference

An appreciation of the value of Bayesian inference is increasing, as have the number and breadth of its applications. However, with this use of the Bayesian calculus comes the potential risk of contamination by ID. In some environments, such as those with undistortable data, ID can never taint Bayesian inference. However, in other likelihood revision tasks, a recognition of the possible presence of ID may improve the accuracy of Bayesian inference or at least prevent its misapplication.

The increased use of Bayesian inference/methods prompts a consideration of where ID might infect such applications. Three such areas are considered, with no claim to completeness or even representativeness. These are: the forecasting by experts of unique, complex events; the prediction of consumption behaviors from past consumption-related data; and Bayesian approaches to statistical inference. In all three cases, likelihood estimates based on new data/information are essential.

### Forecasting by experts

Although Bayesian methods for likelihood revision have generally not been used where only human judgments can provide a numerical evaluation of a datum, their use is increasingly likely. Consider forecasting, a professional task that has achieved recent success with the identification of “superforecasters” (Mellers et al., 2014, 2015). In the forecasting task, a datum is nearly always a unit of complex information. For instance, if the forecasted event is the reelection of Donald Trump in 2020, a positive datum might be the negotiated end to the Korean conflict of 1950-53. In contrast, a negative datum might be the criminal conviction of one of his inner circle. To apply Bayesian inference, forecasters would have to provide not only an explicit prior probability, as they often do now, but also a numerical judgment of the impact of each new datum, something not routinely required. The Bayesian calculus would then yield the revised posterior probability. In such forecasting, the risk of ID would emerge when experts' commitment to a preferred event, such as for or against Trump's re-election, biased their evaluation of a new datum (Mandel, 2008).

One familiar forecasting challenge is estimating the likelihood of the success of a new technology^2^. However, what if the experts who must estimate the technology's success are also biased by ID? Consider the example of drug discovery, where pharmaceutical executives must decide whether to pursue the very expensive process of drug development and governmental approval. One of their challenges is that the only credible source of the likelihood of success is the expert scientists who developed the drug. Because they are often committed to its success, they may bias upward their estimated likelihood of eventual success. Yet to whom else can the decision-making executives turn for an informed likelihood of that success?

As typically practiced now, a forecaster need not provide an explicit likelihood ratio for a datum. If that were required, would it reveal a measurable effect of the prior on the evaluation of the datum in the form of ID? That is, might persistent ID in the human experts undermine the superior accuracy of the Bayesian calculus?

### Prediction of consumption

Bayesian models have a substantial history in commerce (e.g., Erdem and Keane, 1996). More specifically, their use by market researchers relies on both past consumption and internet product search to predict future consumption and, more recently, further search (e.g., Ching et al., 2013; Fong, 2017). However, even as these “consumer learning” models become more sophisticated, they tend to be revised between purchases only by the knowledge of what consumer has searched. The evaluation of that acquired information, along with the possible presence of ID, is not included in the models. How much might the predictive accuracy of such models be improved if they accounted for the biasing influence of ID?

### Statistical inference/analysis

A third and growing domain/area of application of the methods of Bayesian inference is statistical tests of scientific hypotheses. See, for example, the set of papers introduced by Vandekerckhove et al. (2018). As has become well established, using the results from current data to determine when to stop collecting additional data (data-dependent optional stopping) risks invalidating the *p*-values and confidence intervals of classical hypothesis testing. This risk of invalidation has prompted the shift to pre-registering the plan of data collection. Advocates for Bayesian methods claim the elimination of such risks. This claim, in turn, requires the absence of bias in experimenter judgments during the process of inference from collected data.

An analysis of what researchers actually do suggests that this claim may be too strong. For instance, Dunbar (1995, 1999) observed the discussions of research biologists. He found that among the first potential explanations for anomalous data was error in the data collection method (instead of the invalidity of their proffered hypotheses). Surely the same reaction is plausible in the experimental social sciences where data are frequently direct responses from human subjects. Thus, once experimenters who are committed to one hypothesis must judge the validity of their own data, ID may occur.

## The rationality of Bayesian inference vs. the multiple goals of cognitive processing

The Bayesian calculus belongs to the dominant class of decision theories that rely on the unbiased evaluation of information. Indeed, who would want such a theory if it accommodated rather than rejected a bias like ID? Nonetheless, such theories exist, albeit with descriptive rather than normative status. The most relevant may be connectionist models, which not only accept the ID bias but seem to need it. In these models, new information exerts a bidirectional influence on an existing network of related beliefs. A bidirectional process enables them to accommodate ID as the natural (to these models) influence of a current belief on the evaluation of new information (Holyoak and Simon, 1999; Glöckner and Herbold, 2011). This bidirectional influence contributes to the desired goal of a more coherent and stable system of beliefs as it accommodates to the new information.

Connectionist models do not claim rationality. Nonetheless, the goals of internal coherence and network stability are desirable outcomes of the processing of new information. Thus, an undesirable bias like ID becomes necessary to achieving the desirable ends of coherence and stability (Engel and Glöckner, 2013). Nonetheless, the prominence of connectionist models has tended to obscure the situations where coherence pays the price of tolerating biases like ID.

The descriptive value of connectionist theories coupled with the appeal of the goals that they achieve stands against the normative value of the Bayesian calculus. In tasks where that calculus is needed, such as forecasting, the admittedly desirable goals that drive connectionist dynamics must be sacrificed. Instead, techniques of cognitive engineering need to be developed to counter the natural associative mechanisms that yield ID and other phenomena that compromise the Bayesian calculus. Such help may be found in the techniques that enable superforecasting, such as structured methods for eliciting uncertainty estimates and for statistical reasoning. Two promising examples of structured methods for elicitation are the CHAMPS KNOW training that Mellers et al. (2014) used in the IARPA ACE tournament and Mandel (2015) training of intelligence analysts in Bayesian reasoning using natural sampling trees. Also valuable are disconfirmatory challenges from multiple individuals. When individuals work alone to predict an event of only personal relevance, the techniques of superforecasters may have limited application. Nonetheless, when a task is important enough, such as predicting the success of new technologies like drugs, a team may apply the multiple techniques of superforecasting to achieve Bayesian rationality.

## Author contributions

The author confirms being the sole contributor of this work and approved it for publication.

### Conflict of interest statement

The author declares that the research was conducted in the absence of any commercial or financial relationships that could be construed as a potential conflict of interest.

## Footnotes

^1^The optimality of the Bayesian calculus presumes a fixed sample space of possible outcomes and their probabilities. In many real situations, this assumption is violated as, over time, additional outcomes become recognized or the probabilities of the original outcomes are altered (Baratgin and Politzer, 2006, 2010). The present claim of Bayesian optimality excludes such changes in the probability space. Respecting the difference between the effect of information to change the probabilities in a stable space and those that change the space itself, we use Bayesian revision for the former and reserve the term updating for the latter.

^2^By the definition of “new,” there are no baserates to provide historical evidence of the technology's success. The absence of such baserates precludes the tactic of taking an “outside” view as advocated by Lovallo and Kahneman (2003).
